# Challenging diagnosis of prune belly syndrome antenatally: a case report

**DOI:** 10.1186/s13256-019-2120-x

**Published:** 2019-06-29

**Authors:** Waleed H. Alkhamis, Sahar Hassan Abdulghani, Amer Altaki

**Affiliations:** Department of Obstetrics and Gynecology, College of Medicine, King Saud University, King Khalid University Hospital, King Saud University Medical City, P.O BOX 4663, 11412 Riyadh City, Almohammadiyah Kingdom of Saudi Arabia

**Keywords:** Abdominal distention, Clubfeet, Urinary bladder enlargement, Prune belly syndrome

## Abstract

**Background:**

Prune belly syndrome is a rare congenital condition of uncertain etiology.

It is characterized with a triad of abdominal distension due to deficient abdominal wall, genitourinary tract anomalies, and musculoskeletal anomalies. This condition varies in its severity which makes diagnosis challenging during early antenatal scanning.

**Case presentation:**

We reported a severe phenotype of prune belly syndrome which was not fully suspected in a 29-year-old Saudi woman was G4T2P0A1L2 at 21 weeks of gestation at the time of early antenatal presentation; however, it became apparent during diagnosis at a subsequent follow-up scan during advanced gestational age.

**Conclusion:**

We conclude that suspicion of such anomalies through an early antenatal scan require an urgent further follow-up scan in a tertiary center. The referral to the tertiary center must be to an experienced ultrasonographer and maternal–fetal medicine specialist for a decision to be made antenatally regarding the course of pregnancy and post-delivery management based on the severity of the condition.

## Background

Prune belly syndrome (PBS) is known as Eagle–Barrett Syndrome or Obrinsky syndrome and is characterized by a lack of development of abdominal wall muscles giving the appearance of thin wrinkled skin which appears “prune-like” [1, 2], skeletal anomalies, and renal anomalies such as dilated bladder, megaureters, and bilateral cryptorchidism [3]. The exact etiology of this disorder is not known but some studies have indicated that there is a possibility of genetic inheritance and possible chromosomal association with Edward and Down syndrome [3, 5]. More than 95% of affected cases are of male gender [3]. In the USA, PBS affects 3.8 newborns per 100,000 live births [4].

The purpose of this case report is to indicate that PBS is variable in presentation based on the severity of the condition; a close follow-up with complete workup is essential for an antenatal plan of management.

## Case presentation

The authors report a case of a 29-year-old Saudi woman who was G4T2P0A1L2 at 21 weeks of gestation. She was free from medical illness and she had had no previous surgical procedures. She is a housewife; she never smoked tobacco or drank alcohol, and she had no history of recent travel to endemic or pandemic areas. She was referred based on an antenatal ultrasound finding that showed multiple fetal anomalies. This ultrasound had been conducted at another hospital for evaluation and management. Her past obstetrical history was uneventful with two normal term vaginal deliveries and a history of first trimester unexplained miscarriages. She is married to a first-degree cousin working in a governmental institute; there is no history of genetic or congenital anomaly in either of their families.

Her current pregnancy was spontaneous with no history of illicit drug use or exposure to infection or radiation. Her initial early antenatal scan diagnosis showed suspicion of possible fetal diaphragmatic hernia and required further validation which was not possible at the maternal–fetal medicine (MFM) unit at the hospital which also did not have available sonographic specialists. During her first antenatal visit at 21 weeks + 0 day of gestation, the results of her anatomy scan revealed a single viable fetus with estimated fetal weight (EFW) on 50th percentile with normal biometry measurements.

**Fig. 1:**
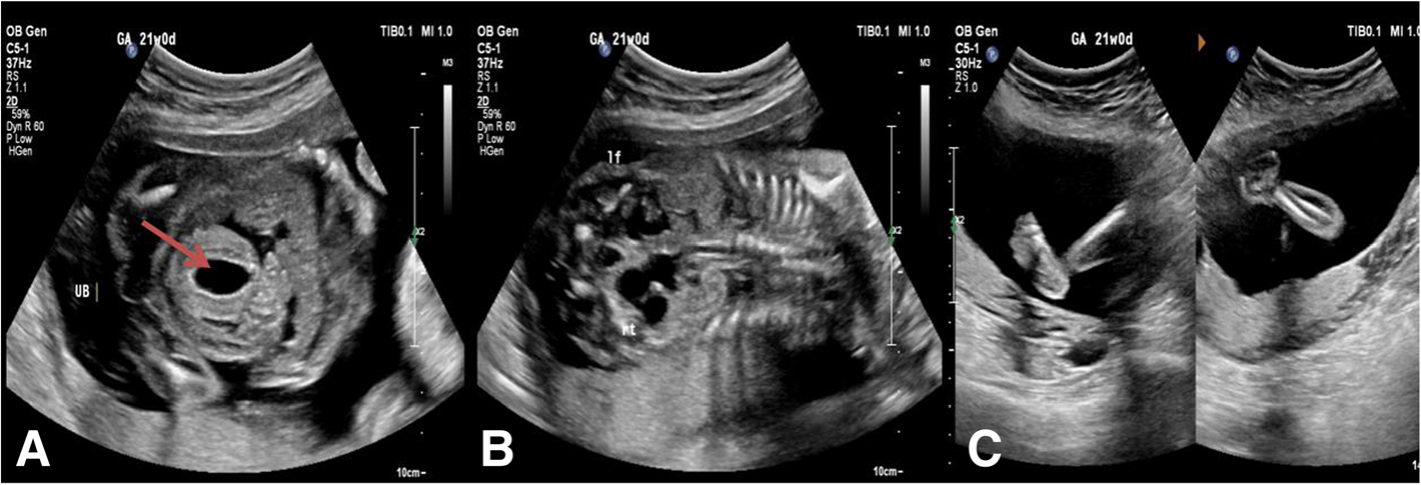
Three images of the first anomaly scan at 21 weeks of gestation in which: **a** an axial view of two-dimensional ultrasound shows fetal distended urinary bladder wall; **b** a coronal view of two-dimensional ultrasound shows distended ureters; **c** an axial view of two-dimensional ultrasound shows bilateral clubfeet. Red arrow is pointing to the fetal urinary bladder

Further detailed anatomy scan findings revealed a male fetus with both kidneys appearing small in size, hyperechoic dysplastic, both ureters were dilated, urinary bladder looked abnormal in shape with thickened bladder wall, and umbilical cord at fetal insertion side appeared thickened. In addition, the diaphragm was seen clearly separating the chest from the abdominal compartments with no evidence of diaphragmatic hernia. Both feet were clubbed and open hands were seen with no other anomalies or any soft marker seen (see Fig. 1.) Based on the multiple fetal structural anomalies discovered, the couple was counseled about the scan findings and advised for further workup, such as: perinatal invasive testing; toxoplasmosis, other (syphilis, varicella-zoster, parvovirus B19), rubella, cytomegalovirus, and herpes (TORCH) screening; and fetal echocardiogram to exclude syndromic or chromosomal causes. This would support reaching a better diagnosis and allow for further discussion on the options available such as the continuity of the pregnancy or termination based on the severity of the fetal condition (see Table 1).

**Table 1:** Timeline

Dates	Relevant past medical history and interventions		
06/09/2018	A 29-year-old Saudi woman, married to a first-degree cousin, was G4T2P0A1L2 at 21 weeks of gestation. She was not known to have any medical illness; she was referred due to suspension of diaphragmatic hernia for further workup		
Dates	Summaries from initial visit and admission follow-up	Diagnostic testing	Interventions
06/09/2018	Presented to the clinic with medical report of findings of suggestive diaphragmatic hernia for further workup to exclude anomalies	On the same day, prenatal screening testing was done and urgent anatomy scan was done that showed genitourinary system anomalies	Patient admitted for amniocentesis and fetal echocardiogram
06/09/2018	The patient was admitted for investigations	Fetal echocardiogram showed no obvious anomalies	Amniocentesis done and sample sent to the laboratory
07/09/2018	The patient discharged with clinic follow-up	Negative result of prenatal infections	
20/10/2018	The patient did not come to her clinic follow-up	Result of amniocentesis was normal	
19/11/2018	Patient came to the clinic after the department coordinator called her	Repeated ultrasound scan showed severe and progressing anomalies compared to the first scan. Counseling done regarding these findings	Patient admitted to the ward
19/11/2018	Patient was in the hospital for observation and tapping of the bladder. The case was presented in a multidisciplinary team meeting with perinatologist and neonatologist		The meetings revealed no intrapartum management in labor with no fetal monitoring and caesarian section would be preserved for maternal indication. Post-delivery plans not to resuscitate the infant and applying comfort care post-delivery were also explained
20/11/2018	The patient received extensive counseling regarding the outcomes of the meeting and proper plan		Tapping of bladder performed and samples sent to whole exome sequencing
21/11/2018	Patient delivered with no complications, baby boy with prune belly syndrome features, he died within 2 hours		
22/11/2018	Patient counseled regarding the future plan of pregnancy management then discharged		
06/12/2018	Normal placental histopathology, whole exome sequencing result was not obtained due to laboratory error

**Fig. 2:**
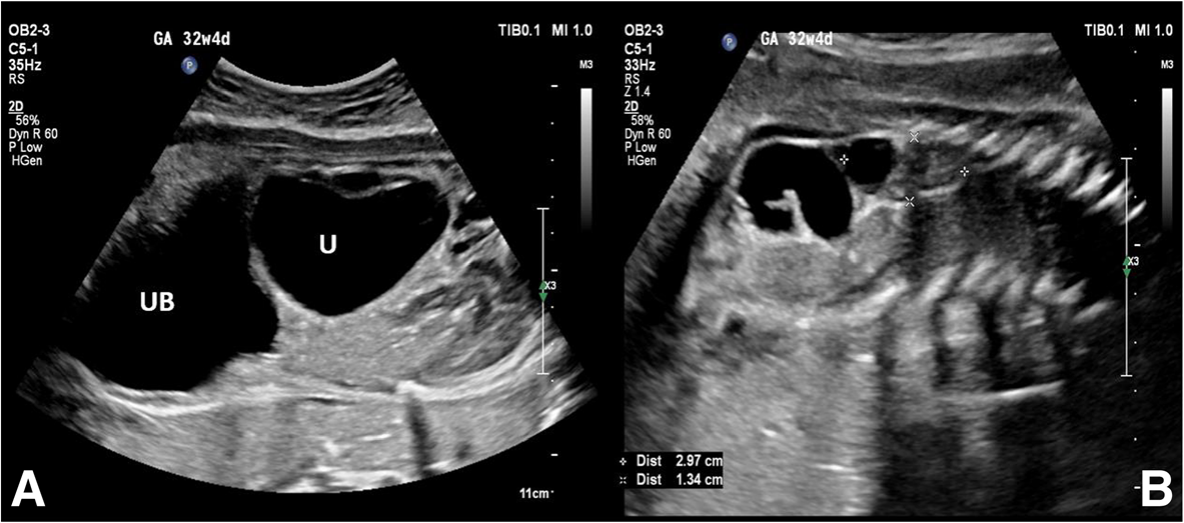
Two pictures of ultrasound at 32 weeks and 4 days of gestation in which: **a** an axial view of two-dimensional ultrasound shows (*UB*) progressive and severe enlargement of urinary bladder, and (*U*) progressive enlargement of ureter; **b** a coronal view of two-dimensional ultrasound shows a hugely distended abdomen and anhydramnios. *U* ureter, *UB* urinary bladder

Our patient had some social issues and was also following her condition in another institute and only revisited our center at 32 weeks and 4 days of gestation. At our center another follow-up scan revealed a single viable fetus, cephalic in presentation, anhydramnios with normal head and femoral length biometry. Unfortunately, the abdominal circumference (AC) was not taken due to the extremely distended abdominal wall that prevented any further visualization by ultrasound. The right kidney measured 3.4 × 1.1 cm with a small cyst, the left kidney measured 2.9 × 1.3 cm with bilateral hugely dilated ureter and urinary bladder (mega cyst) (see Fig. 2).

### Other test results

TORCH screen test results were non-reactive. Amniocentesis was performed and showed normal chromosomal results. A fetal echocardiogram allowed for limited examination due to anhydramnios; however, no obvious cardiac anomalies were noted. Lungs appeared compressed due to severely distended abdomen from the progressively enlarged urinary system, otherwise no other abnormal findings noted. The couple was counseled by the MFM team about the worsening condition from the recent scan findings and were told about the poor fetal prognosis and the high mortality rate, secondary to severe lung compression with the presence of anhydramnios which would lead to lung hypoplasia and cause fetal demise.

It was explained that the entire urinary system was affected with severe dilatation causing severe abdominal wall dilatation and for this reason measuring fetal AC had been difficult antenatally. Options were discussed with the couple:

- Termination of pregnancy to avoid obstetrical complication during labor which is fetal abdominal dystocia as it was difficult to measure the abdominal wall antenatally with the severe progressive renal system dilation with advancing gestational age versus
- To wait until term pregnancy while knowing the poor fetal prognosis

Furthermore, antenatal interventions were offered to the couple including tapping of the fetal bladder and ureters prior to induction of labor and to then send the amniotic fluid sample for further genetic testing. Our patient’s case was initially discussed by a multidisciplinary team which included a perinatologist and a neonatologist before finally making a combined agreement and alignment with the couple who decided to terminate the pregnancy; a caesarian section would be preserved for maternal indication and comfort care post-delivery to born infant were also explained.

At 32 weeks and 5 days of gestation, tapping of the fetal bladder and ureter was performed and samples of amniotic fluid were sent for whole exome sequencing (WES) test; however, unfortunately, after waiting a few weeks for the results, no results could be determined due to a laboratory error.

**Fig. 3:**
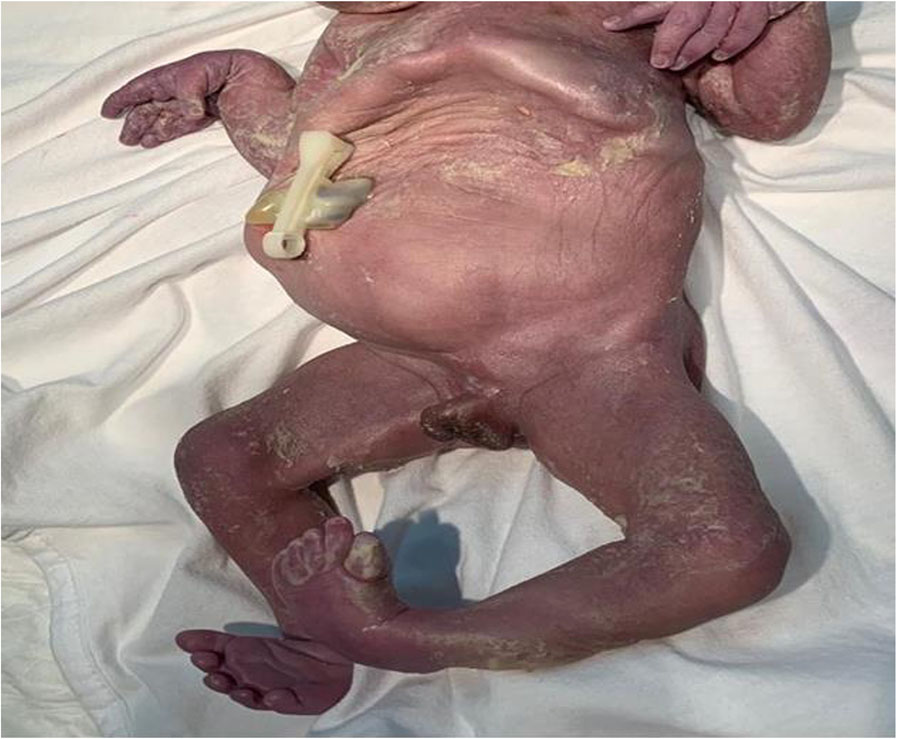
A neonate born with distended abdomen, absent abdominal musculature, thin wrinkled skin, cryptorchidism, clubfeet, and clenched hands

Our patient underwent induction of labor to terminate the pregnancy and delivered vaginally a male neonate with Apgar score of 2 in 1 minute and 5 in 5 minutes, weighing 1800 grams without any complications. The vital signs revealed blood pressure of 90/60, pulse 100 beats /minute, and temperature of 36 °C. Clinical examination of the newborn revealed distended abdomen and thin wrinkled skin, retracted chest, cryptorchidism, and clubbed feet; no facial anomalies were noted and the features were most likely to be suggestive of PBS (see Fig. 3*)*. The newborn died 2 hours post-delivery.

The placenta was sent for a histopathology examination as a part of the workup and the result revealed normal findings.

A postmortem examination was not offered to the couple since this is not conducted in the center. The couple was counseled prior to discharge regarding future pregnancy plans, despite low reoccurrence. It was also highlighted to them the importance of having early prenatal testing in a center in which there were well-trained sonographers and a high risk in pregnancy unit available. They were also informed about the lack of result of WES test due to laboratory error and they were fine.

## Discussion and conclusions

We presented a severe phenotype of PBS diagnosed antenatally with large distended abdomen and genitourinary system anomalies; after counseling, our patient underwent antenatal intervention and tapping of bladder and ureters to prevent obstetrical complications and terminated the pregnancy.

PBS is a rare congenital anomaly characterized by anomalies of genitourinary and musculoskeletal systems in which other systems could be involved [3]. Features can be identified antenatally during the first trimester. Some case series and reports indicated that early detection can be achieved between 10 and 13 weeks of gestation where the result of this early presentation is due to an obstruction or stricture of urinary tract system [6]. Researchers who conducted a review of the literature identified 26 cases of PBS antenatally and among these cases 23 presented with urinary tract anomalies; the fetal mean age at diagnosis ranged between 12 and 22 weeks with no siblings affected with PBS [6].

A recent literature review concluded diagnosis of PBS can be made based on identification of distended bladder and possible presentation of hyperechogenic kidneys. [7]. Prognosis of PBS syndrome varies depending on time of presentation, nature, and phenotype of accompanying anomalies, but the early detection of PBS features in ultrasound were associated with very poor prognosis such as stillbirth [7].

In addition, prognosis of PBS is highly dependent on lung hypoplasia and severity of renal anomalies, in which lung maturity will determine the prognosis in the neonatal period and renal function will determine long-term prognosis and outcomes [8, 9].

In this case report, prenatal ultrasound was able to detect abnormalities of the urinary tract associated with the typical appearance of the abdominal wall but postnatal diagnosis of PBS can be easily established. In our case, the initial reason for referral was to confirm the diagnosis based on multi-systems involvement with anomalies. The presence of dilated urinary bladder and clubfeet with normal AC needed further workup to exclude syndromic or chromosomal causes. The diagnosis of PBS was not highly suspected with such a presentation; however, our patient also followed up with other modalities such as fetal echocardiogram and ultrasound which confirmed the diagnosis. During advancing gestational age, features of PBS including progressive and severe dilation of urinary bladder and ureters, and abdominal distention due to absence of abdominal muscles became more obvious.

The initial presentation and ultrasound diagnosis were not conclusive toward PBS as a diagnosis until severe distension in fetal AC appeared during advancing gestational age. Managing such a condition required some time to gather the full information for decision making. This case is a rare type of congenital disorder and it is also considered unusual in its presentation. The challenges in approaching the diagnosis of PBS based on the severity of the condition subsequently played a major part in antenatal counseling and management. Not all multiple fetal anomalies are lethal and the prognosis varies based on the severity of pulmonary hypoplasia and urinary tract abnormalities for which an extensive investigation is required and advised before discussion about termination of pregnancy.

The lessons obtained from this presentation are that termination of pregnancy is a very challenging and difficult decision to make in the presence of multiple fetal anomalies, a complete workup and detailed counseling are required assuring the survival rate based on the severity of the condition. In conclusion, PBS is a rare entity worldwide with wide variability in severity and clinical manifestations. It presents a spectrum of features that may be detected during early antenatal ultrasound and hence requires an experienced sonographer in a tertiary center and referral to MFM specialists for extensive counseling and management plan.

## Data Availability

All data underlying the results are available as part of the case report and no additional source data are required.
